# Integrated physiological and transcriptomic analyses reveal MT-mediated aluminum tolerance mechanisms in celery

**DOI:** 10.1186/s12870-026-08994-w

**Published:** 2026-06-03

**Authors:** Zheng Guo, Gongkai Qiu, Xiaohan Lu, Junting Liu, Peiran Chen, Qiuxia Li, Xinyu Zhou, Hu Wang, Fenfen Luo, Zhiyuan Liu, Fengyun Lei, Mengyao Li, Chengyao Jiang, Wei Lu, Yangxia Zheng

**Affiliations:** 1 College of Horticulture, Sichuan Agricultural University, Chengdu, 611130 China; 2 Agricultural Equipment Research Institute, Chengdu Academy of Agricultural and Forest Sciences, Chengdu, 611130 China

**Keywords:** MT, Aluminum Stress, Celery, Transcriptomic

## Abstract

Aluminum toxicity is one of the primary stresses limiting plant growth in acidic soils, mainly through the induction of oxidative damage and disruption of hormone-mediated developmental pathways. Melatonin (MT), a critical endogenous plant regulator, has been demonstrated to enhance plant tolerance to various abiotic stresses; however, its molecular mechanisms in alleviating aluminum stress remain to be further elucidated. In this study, celery (*Apium graveolens* L.) was used as the experimental material, and physiological phenotype analysis combined with transcriptome sequencing was performed to systematically explore the alleviative role of MT under aluminum stress and its molecular basis. The results showed that aluminum stress significantly inhibited the growth and development of celery, whereas melatonin treatment markedly promoted biomass accumulation and root growth, thereby effectively alleviating the growth inhibition caused by aluminum toxicity. Specifically, MT treatment significantly increased plant height (by 6.7%) and root activity (by 250.9%) while reducing oxidative damage, as evidenced by decreased H_2_O_2_ content (by 25.1% and 21.9%, respectively). Transcriptome analysis revealed that aluminum stress markedly induced phenylpropanoid and flavonoid biosynthesis pathways, whereas MT treatment enhanced the expression of genes involved in glutathione metabolism, particularly with the significant upregulation of *GST* family genes. In addition, MT treatment affected multiple plant hormone signaling pathways (such as auxin, brassinosteroid, and salicylic acid), accompanied by changes in the expression of key genes, including *ARF*, *GH3*, *SAUR*, *TCH4*, *CYCD3*, and P*R-1*. Overall, melatonin treatment is closely associated with the regulation of antioxidant metabolism and plant hormone signaling pathways and may alleviate the inhibitory effects of aluminum toxicity on celery growth. This study provides a theoretical basis for the further application of melatonin to enhance aluminum tolerance in crops.

**Supplementary Information:**

The online version contains supplementary material available at 10.1186/s12870-026-08994-w.

## Introduction

 Celery (*Apium graveolens* L.) is an important biennial vegetable crop belonging to the Apiaceae family and has been widely cultivated for both culinary and medicinal purposes [1]. Owing to its high contents of vitamins [2], flavonoids, and aromatic compounds, celery is increasingly valued as a functional vegetable with recognized health-promoting properties [3]. With the expansion of protected cultivation and intensive agricultural practices, maintaining stable celery production under adverse soil conditions has become an important agronomic concern.

Soil acidification, driven by acid rain, excessive fertilization, and industrial activities, has emerged as a major constraint on crop productivity worldwide [4]. Under acidic conditions (pH < 5.0), aluminum is solubilized into phytotoxic forms, predominantly Al^3+^ and AlOH^2+^ species [5–6], which severely inhibit plant growth. Aluminum toxicity primarily targets root systems, resulting in suppressed root elongation, impaired nutrient uptake, and disrupted cellular homeostasis. The binding of Al^3+^ to the cell wall is one of the key mechanisms underlying aluminum toxicity. Aluminum ions bind to components such as pectin and hemicellulose in the cell wall, increasing the rigidity of the cell wall and inhibiting cell expansion and growth. The formation of this physical barrier obstructs root elongation, thereby affecting water and nutrient uptake by the plant [7]. Additionally, aluminum stress induces the deposition of callose in the roots, and plants accelerate the accumulation of mucilage in the root tip area under aluminum stress, forming a mechanical barrier that further restricts root elongation and interferes with nutrient absorption [8]. To cope with aluminum toxicity, plants have evolved two major tolerance mechanisms: external exclusion and internal tolerance [9]. Plants can restrict the entry of Al^3+^ into cells through cell wall immobilization and organic acid secretion, whereas internal detoxification is achieved via vacuolar sequestration and endogenous chelation. These processes are regulated by multiple aluminum tolerance–related genes, forming a complex adaptive response network.

Melatonin (*N*-acetyl-5-methoxytryptamine) is an indoleamine molecule ubiquitously distributed in living organisms [10]. In plants, MT is synthesized via a tryptophan-dependent pathway and has been recognized as an important regulatory molecule involved in growth, development, and stress adaptation [11]. Accumulating evidence demonstrates that MT plays a central role in modulating root architecture [12], photosynthetic performance [13], and antioxidant capacity [14]. In addition to its well-documented involvement in plant defense against pathogens [15–17]. Studies have also shown that melatonin enhances plant tolerance to abiotic stresses [18], including heavy metal toxicity [19], high temperature [20], salinity [21], and oxidative stress [22], largely through the maintenance of redox balance and regulation of stress-responsive gene expression.

In recent years, the role of melatonin in alleviating aluminum stress in plants has received increasing attention. Previous studies have shown that exogenous melatonin primarily enhances plant tolerance to aluminum toxicity by strengthening the antioxidant defense system, reducing reactive oxygen species accumulation, and maintaining cellular redox homeostasis [23]. In addition, melatonin can mitigate the inhibitory effects of aluminum stress on plant growth by regulating carbon and nitrogen metabolism as well as growth-related physiological processes [24]. Despite these advances, the potential role of melatonin in alleviating aluminum-induced stress has not been sufficiently explored, particularly in economically important vegetable crops such as celery, for which systematic studies remain limited. Moreover, current research on melatonin-mediated aluminum tolerance has largely been confined to individual physiological or biochemical aspects, and the key regulatory networks underlying the coordination of multiple stress-response pathways at the transcriptomic level for mitigating aluminum stress remain unclear. Based on this, it is hypothesized that melatonin may primarily function as a signaling molecule, alleviating aluminum-induced damage in plants by regulating the antioxidant defense system and related hormone signaling pathways and coordinately modulating multiple stress-response processes at the transcriptional level. Therefore, this study used the celery cultivar “Oster Ziyu Xiangqin” as the experimental material to systematically evaluate the effects of exogenous melatonin on growth and physiological characteristics under aluminum stress. In combination with transcriptome sequencing analysis, the potential molecular regulatory mechanisms were elucidated, thereby providing a theoretical basis for improving aluminum tolerance in celery production systems.

## Materials and methods

### Experimental materials and environment

The celery cultivar selected for the experiment was ‘Oster Ziyu Xiangqin’ (Haride Seed Co., Ltd.; Hebei, China). The reagents included aluminum sulfate octadecahydrate (Al_2_(SO_4_)_3_·18H_2_O; Chengdu Kelong Chemical Co., Ltd.; Sichuan, China) and MT (Beijing Solarbio Science & Technology Co., Ltd.; Beijing, China).

The experiment was carried out in 2024 at a plant factory facility of the Chengdu Academy of Agriculture and Forestry Sciences, Chengdu, Sichuan, China (103°51′E, 30°42′N). The experimental conditions were as follows: a light intensity of 400 ± 10 µmol·m^− 2^·s^− 1^, a photoperiod of 12 h/12 h, a temperature of 25 °C, a relative humidity of 50%–60%, and a CO_2_ concentration at ambient atmospheric levels.

### Experimental treatment

Uniform and healthy celery seeds at full maturity were chosen and germinated on moistened cloth in a constant-temperature incubator set at 22 °C. Once germination reached approximately 90%, the seedlings were transferred to culture trays for subsequent hydroponic growth. After the seedlings developed two fully expanded true leaves, they were maintained in 1/3 Hoagland nutrient solution, with the nutrient solution adjusted to a pH of 6.5 ± 0.5 and the EC (Electrical Conductivity) was 1.0 ± 0.1 mS·cm^− 1^. When celery seedlings developed four leaves, they were transferred to hydroponic trays for acclimation.

The concentrations of aluminum and melatonin were determined based on preliminary screening experiments (Supplementary Material 1.). Treatments were initiated after 20 d of acclimation, a total of three treatments were established, each with three replicates: (1) CK (0 µmol·L^− 1^ Al_2_(SO_4_)_3_·18H_2_O), (2) Al (800 µmol·L^− 1^ Al_2_(SO_4_)_3_·18H_2_O), and (3) MT (150 µmol·L^− 1^ MT + 800 µmol·L^− 1^ Al_2_(SO_4_)_3_·18H_2_O). For the Al treatment, the weighed solid aluminum sulfate was directly added to the nutrient solution and mixed thoroughly, with the treatment applied every 5 d. MT was applied by foliar spraying until droplets began to drip from the leaf surface, also at 5 d intervals, and the Al treatment was applied simultaneously. Measurements were conducted daily throughout the experiment, and adjust the pH of the solution to 4.5 daily using H_2_SO_4_ and NaOH. Sampling was conducted on day 10 of treatment. For each treatment, leaves and petioles from five randomly selected healthy plants were collected. The collected samples were rapidly immersed in liquid nitrogen and subsequently stored at − 80 °C for later analysis of physiological indices.

### Determination of growth parameters

On the 10th day of treatment, five plants were randomly selected for the measurement of growth parameters. Plant height and root length were measured using a ruler; petiole length and width were measured using a Vernier caliper. The fresh weights of the aboveground and belowground parts were determined using an analytical balance, and their dry weights were measured after drying at 80 °C for 72 h.

For root morphological analysis, the shoots of celery from each treatment were removed, and the roots were washed and scanned using a root analysis system (WinRHIZO Pro LA2400, Canada) to obtain images. Root morphological parameters were then analyzed using WinRHIZO 5.0 software.

Root activity was determined using the α-naphthylamine oxidation method, and the activity was calculated based on the amount of α-naphthylamine oxidized [25].

### Determination of aluminum content

Aluminum content was determined by inductively coupled plasma–mass spectrometry (ICP–MS) [26], and was measured by an external institution (Chengdu Boyangang Biotechnology Co., Ltd.; Chengdu, China).

### Determination of photosynthetic indicators

On the 10th day of treatment, net photosynthetic rate (Pn), intercellular CO_2_ concentration (Ci), stomatal conductance (Gs), and transpiration rate (Tr) were measured in leaves using a portable photosynthesis analyzer (LI-6800, LI-COR Biosciences, Lincoln, NE, USA). Maximum photosynthetic efficiency (Fv/Fm), actual photosynthetic efficiency (Y(Ⅱ)), photochemical quenching (qP), and non-photochemical quenching (NPQ) were measured using a portable fluorometer (PAM-2500, Heinz Walz GmbH, Effeltrich, Germany).

Fresh celery leaf samples were immersed in an acetone–ethanol mixed solution. After all samples were completely decolorized, absorbance was recorded at 470, 663, and 646 nm [27].

### Determination of antioxidant system parameters

The levels of O_2_^·−^ and H_2_O_2_ were quantified using assay kits (Beijing Boxbio Science & Technology Co., Ltd.; Beijing, China). MDA levels were determined using the thiobarbituric acid (TBA) assay [28].

The activities of superoxide dismutase (SOD), peroxidase (POD), and catalase (CAT) were determined according to the methods described by Foad [29], Omran [30], and Gururani [31], respectively, and the results were expressed as U·g^− 1^ .

### Determination of glutathione-related parameters

The levels of GST、GSH and GSSG were quantified using assay kits (Beijing Solarbio Science & Technology Co., Ltd.; Quanzhou, China).

## Determination of hormone levels

The contents of auxin (IAA), brassinosteroids (BR), and salicylic acid (SA) were determined using ELISA kits (Ruixin Biotechnology Co., Ltd.; Beijing, China).

### Transcriptome sequencing

On the 10th day of treatment, fresh leaves from different treatments were collected and rapidly frozen in liquid nitrogen. Total RNA was extracted from pooled sample, and its integrity and quality were assessed. Samples meeting the requirements for transcriptome sequencing (RIN > 8) were then sent to Novogene Bioinformatics Technology Co., Ltd. (Beijing, China) for sequencing on the Illumina NovaSeq X Plus platform, generating 150 bp paired-end reads.

Raw sequencing data (raw reads) contained a small proportion of reads with sequencing adapters or low-quality sequencing reads. After filtering the raw data, clean reads were obtained for subsequent analyses. Read counts were normalized using fragments per kilobase of FPKM, followed by correction for multiple hypothesis testing. Differential expression analysis of read counts was performed using DESeq2 (v1.20.0). Differentially expressed genes (DEGs) were identified using the criteria of |log2FoldChange| ≥ 1 and padj ≤ 0.05. Finally, KEGG pathway enrichment analysis and Gene Ontology (GO) functional enrichment analysis of the DEGs were conducted using the clusterProfiler (version 3.8.1).

### qRT-PCR validation

The extracted RNA was reverse-transcribed into cDNA using a commercial kit (Hangzhou Xinjing Biological Reagent Development Co., Ltd.; Zhejiang, China). Quantitative real-time PCR (qRT-PCR) was performed using the Archimed X4 system (Beijing Kunpeng Gene Science Instrument Co., Ltd.; Beijing, China) and F488 SYBR qPCR Mix reagent (Jiangsu Biosharp Biotechnology Co., Ltd.; Jiangsu, China). Relative transcript abundance was determined using the 2^−ΔΔCt^ [32] method, with Actin serving as the internal reference gene (Supplementary Material 2.).

### Data analysis

Data were organized and statistically analyzed using Excel 2019 and SPSS 27.0.1. Differences among treatments were tested by one-way ANOVA, followed by Duncan’s multiple range test and the least significant difference (LSD) test for significance analysis. Values shown in figures and tables are presented as mean ± standard deviation (SD), with the significance level set at *p* < 0.05. Figures were generated using GraphPad Prism 9.5.

## Results

### Effects of exogenous MT on growth and aluminum accumulation in celery under aluminum stress

**Fig. 1:**
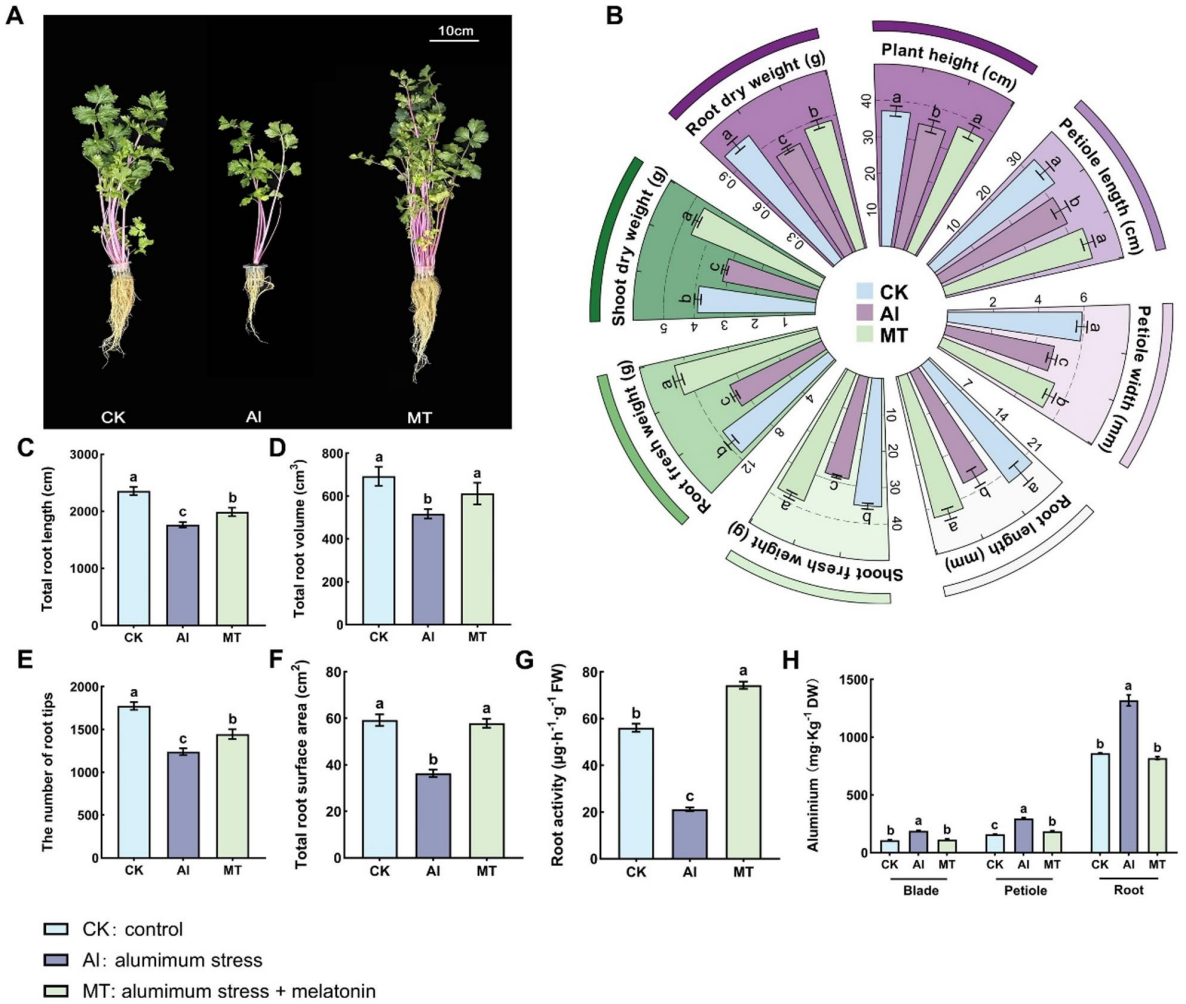
Effects of MT on celery growth, root structure, and aluminum accumulation under aluminum stress. **A** Growth phenotype of celery under different treatments. MT represents the Al + MT treatment, the same below. **B** Growth parameters of aboveground and underground parts. **C**–**F** Root morphological parameters. **G** Root activity. **H**. Aluminum content in different plant parts. Different letters denote statistically significant differences at *p* < 0.05, the same below

As shown in Fig. 1A, celery growth differed significantly among the different treatments. Relative to the CK treatment, the Al treatment significantly reduced plant height, petiole length and width, and root length, and also inhibited the accumulation of fresh and dry weight in both aboveground and belowground parts (Fig. 1B). Additionally, Al treatment markedly reduced root system parameters. (Fig. 1C–F), while root activity (Fig. 1G) was also significantly reduced. These findings indicate that aluminum toxicity affects celery growth at multiple levels, including morphology and biomass accumulation. After MT application, celery growth was significantly improved. Compared to Al treatment, the MT treatment significantly enhanced plant height, petiole morphological parameters, root length, and both aboveground and belowground fresh and dry weight. MT also markedly promoted root elongation and branching, while root activity elevated by 250.9%. Furthermore, after Al treatment, the aluminum content in different parts of celery increased markedly (Fig. 1H), whereas the MT treatment significantly decreased aluminum concentrations across all tissues, exhibiting reductions of 39.5%, 37.5%, and 37.8%, respectively, relative to the Al treatment. Overall, compared with the Al treatment, MT treatment significantly enhanced the growth of celery under aluminum stress, as evidenced by improved plant morphology, biomass accumulation, root architecture, and root activity, accompanied by a substantial reduction in Al^3+^ content across plant tissues.

### Effects of exogenous MT on the photosynthetic characteristics of celery under aluminum stress

**Fig. 2:**
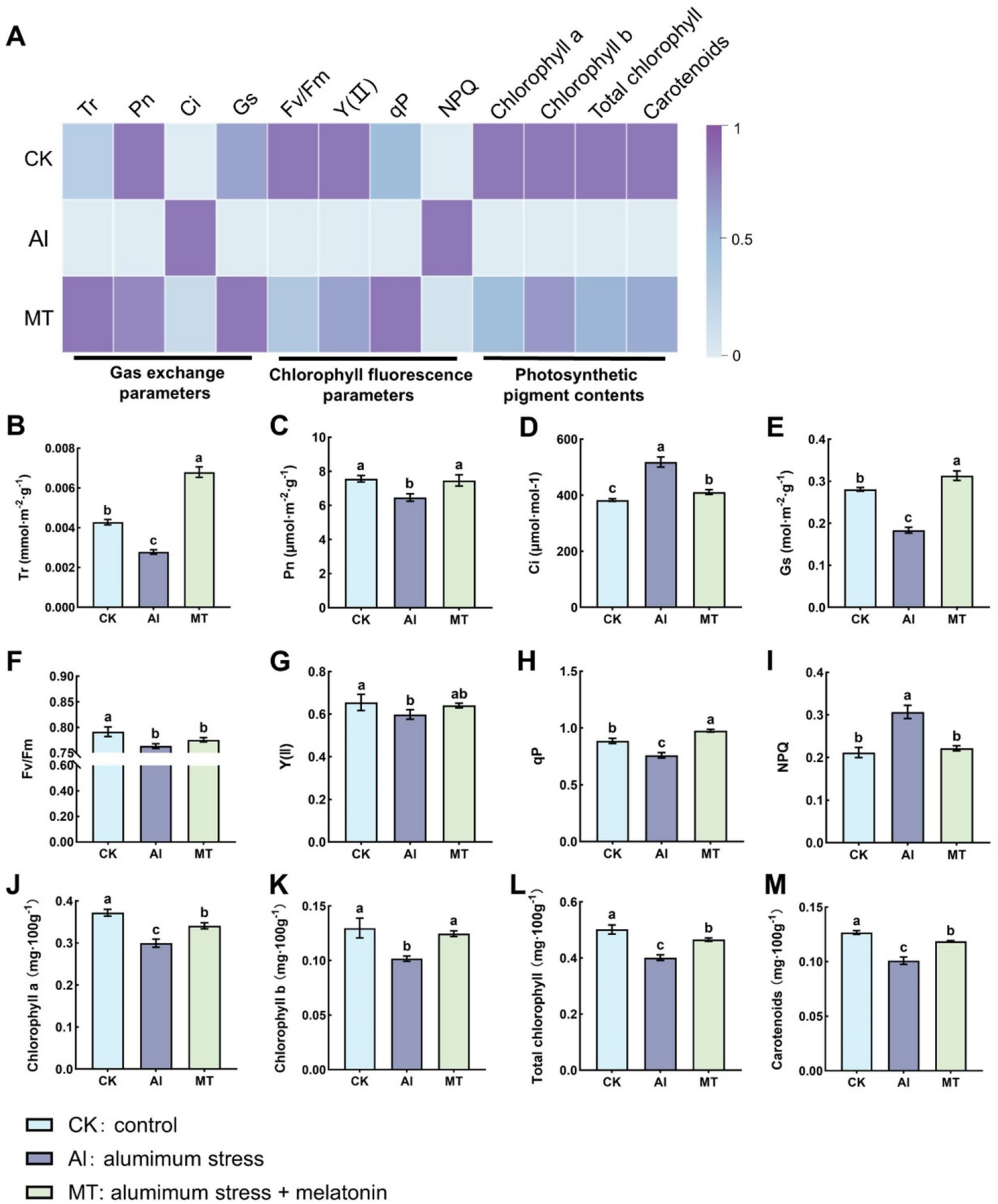
Comprehensive effects of exogenous MT on gas exchange, chlorophyll fluorescence, and photosynthetic pigment content under aluminum stress. **A** Comprehensive photosynthetic effect heatmap, with data normalized. **B**–**E** Gas exchange parameters. **F**–**I** Chlorophyll fluorescence parameters. (**J**–**M**) Photosynthetic pigment content

The trend heatmap (Fig. 2A) demonstrates that aluminum stress led to an overall decline in photosynthesis-related parameters in celery, whereas MT treatment promoted the recovery of photosynthetic performance to levels closer to the CK condition. Compared with the CK, Al treatment resulted in a significant decrease in Tr (Fig. 2B), Pn (Fig. 2C), and Gs (Fig. 2D) in celery leaves, while Ci (Fig. 2E) was significantly increased. This suggests that aluminum stress significantly impaired the photosynthetic carbon assimilation capacity of celery. After MT application, Tr and Gs were markedly improved, increasing by 144.5% and 70.5%, respectively, compared with the Al treatment, while Pn recovered to CK levels and Ci was significantly reduced by 20.7%. These results indicate that MT effectively alleviated aluminum-induced inhibition of leaf gas exchange. Chlorophyll fluorescence parameters further showed that aluminum treatment significantly decreased Fv/Fm (Fig. 2F) and Y(Ⅱ) (Fig. 2G), while also reducing qP (Fig. 2H) and increasing NPQ (Fig. 2I), indicating that aluminum stress impaired the photochemical efficiency of PSⅡ and enhanced energy dissipation. Under MT treatment, these fluorescence parameters were substantially improved, with a notable enhancement of PSⅡ photochemical activity and a recovery trend toward CK levels, indicating that MT can alleviate aluminum-induced impairment of PSⅡ functionality. In addition, Al treatment significantly reduced the contents of photosynthetic pigments (Fig. 2J–M) in celery leaves, whereas MT treatment improved pigment contents by 13.9%, 22.5%, 16.1%, and 17.7%, respectively, compared with the Al treatment, although the levels were still lower than those of the CK treatment. Overall, aluminum stress impacted the photosynthetic performance of celery at multiple levels, including gas exchange, PSⅡ photochemical efficiency, and photosynthetic pigment accumulation. Exogenous MT application was able to alleviate these adverse effects to some extent.

### Effects of exogenous MT on the antioxidant system of celery under aluminum stress

**Fig. 3:**
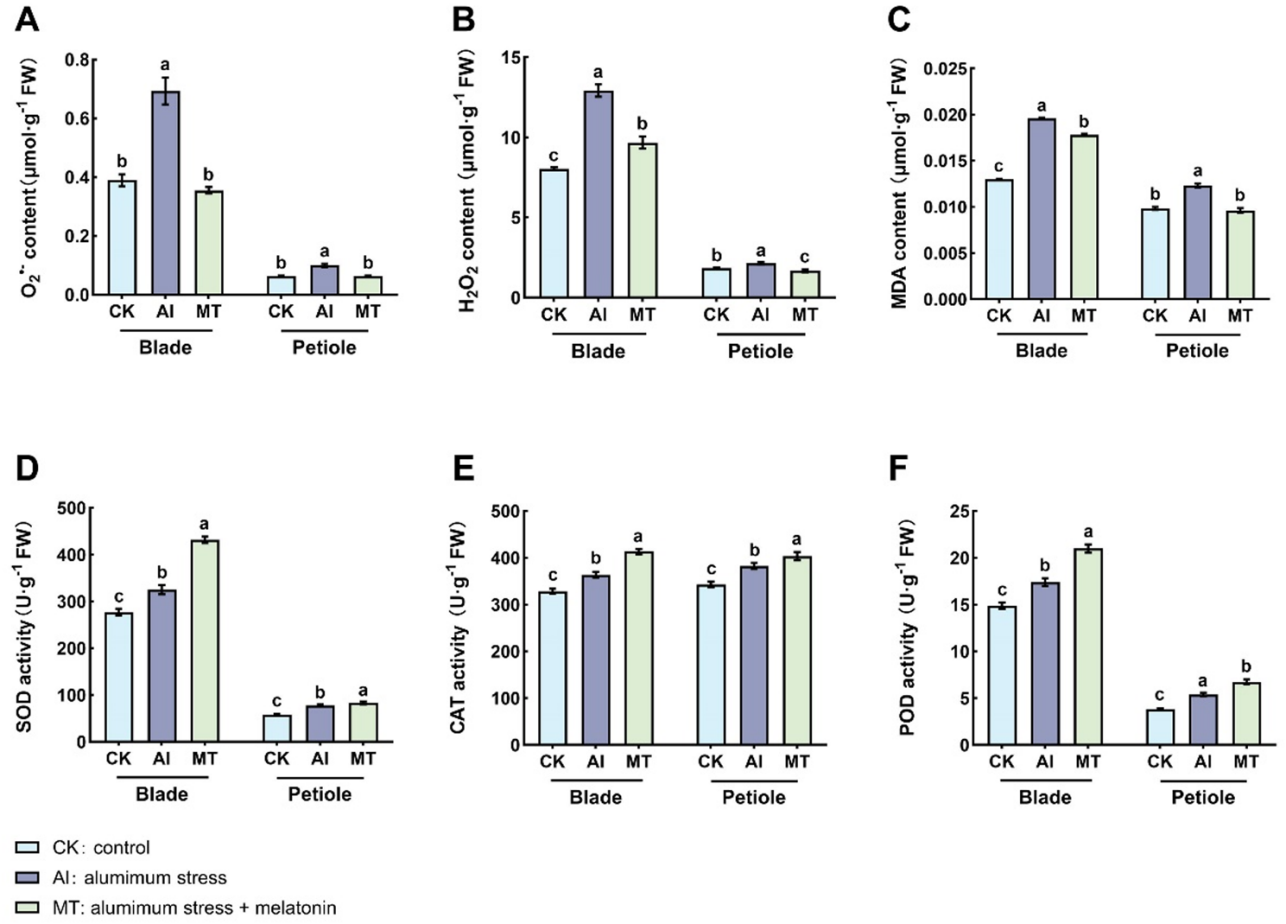
Effects of different treatments on the antioxidant system of celery under aluminum stress. **A** O_2_^·−^ content. **B** H_2_O_2_ content. **C** MDA content. **D** SOD activity. **E** CAT activity. **F** POD activity

Comprehensive changes in antioxidant system–related parameters showed that aluminum stress significantly induced oxidative stress in celery plants. As shown in Fig. 3, compared with the CK treatment, the Al treatment substantially increased the contents of reactive oxygen species (ROS) (Fig. 3A, B) and MDA (Fig. 3C) in leaves and petioles, and also enhanced the activities of antioxidant enzymes (Fig. 3D–F). The results indicate that aluminum stress may disrupt the dynamic balance between the production and scavenging of reactive oxygen species, placing the plants under a certain degree of oxidative stress. Following exogenous MT application, the accumulation of oxidation-related indicators was markedly reduced, whereas the overall activities of antioxidant enzymes were further enhanced. Taken together, MT plays a crucial role in alleviating aluminum stress by enhancing antioxidant defense capacity and reducing oxidative damage, thereby indirectly protecting the photosynthetic system and promoting plant growth recovery.

### Transcriptome sequencing and expression analysis of DEGs

In the transcriptome analysis of the nine samples completed in this sequencing, the Q20 content ranged from 99.29% to 99.36%, the Q30 content ranged from 96.77% to 97.16%, and the GC content ranged from 42.19% to 42.93%. Over 93.40% of the clean reads were mapped to the celery genome (Supplementary Material 3.). Principal component analysis (Supplementary Material 4.) and correlation analysis (Supplementary Material 4.) both showed significant differences among the three treatment groups and high reproducibility within each group.

**Fig. 4:**
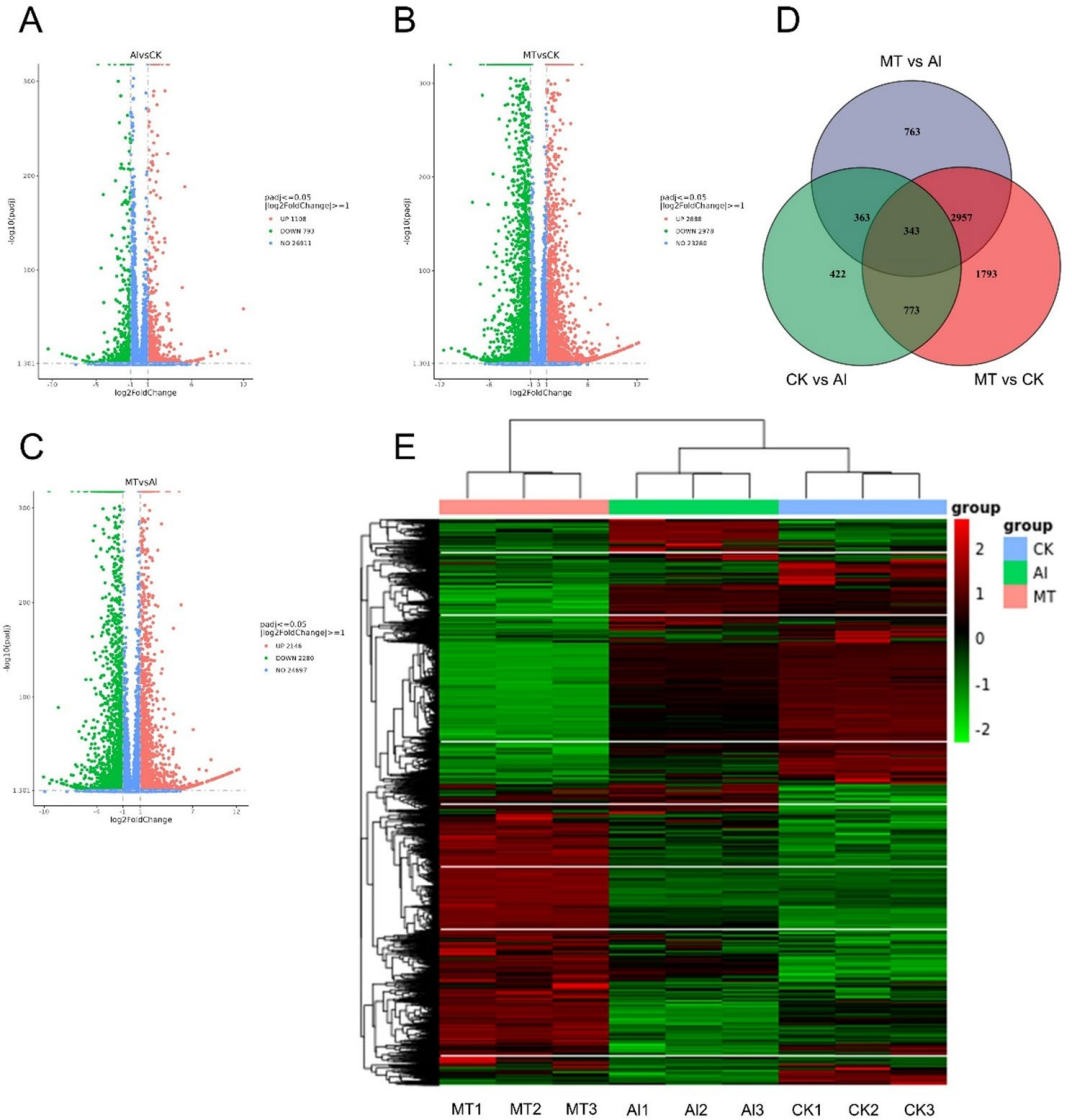
Characteristics of differentially expressed genes. **A**–**C**. Volcano Plot of DEGs. **D**. Venn diagram of DEGs. **E**. Cluster diagram of DEGs

Following gene expression quantification, DEGs were screened based on thresholds of |log_2_FoldChange| ≥ 1 and an adjusted p-value (padj) ≤ 0.05, and the results are summarized as follows. Figure 4A–C show the numbers of DEGs between different comparison groups. Among them, the MT vs. CK comparison contained the largest number of DEGs, with 2,888 upregulated genes and 2,978 downregulated genes. Figure 4D illustrates the intersections of DEGs among multiple treatment comparisons. The results showed that the largest overlap of DEGs (2,957 genes) occurred between MT vs. Al and MT vs. CK, whereas the smallest overlap (363 genes) was observed between MT vs. Al and CK vs. Al. In total, 343 genes exhibited differential expression shared among all three comparison groups, and the numbers of unique genes in each comparison group were 422, 763, and 1793, respectively (Supplementary Material 5.). Hierarchical clustering analysis based on the FPKM values of DEGs is presented in Fig. 4E. The heatmap shows clear differences in clustering patterns among treatments, and gene expression profiles under the CK and Al treatments were more similar to each other.

### qRT-PCR validation

**Fig. 5:**
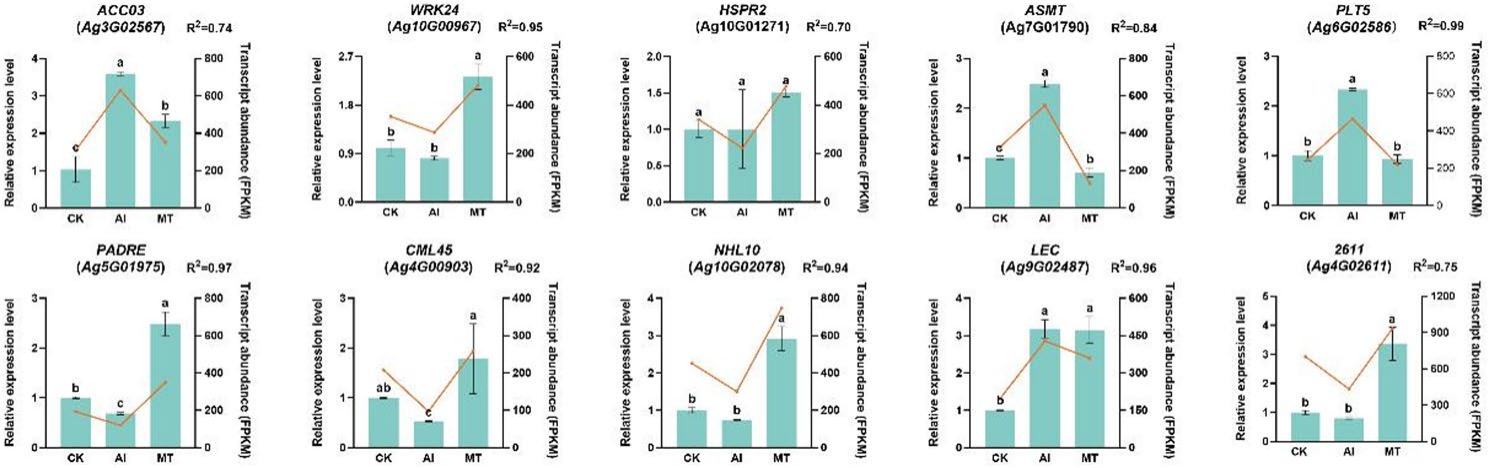
qRT-PCR validation results

The qRT-PCR results shown in Fig. 5 displayed expression patterns that were largely in agreement with those obtained from RNA-Seq, further validating the reliability and accuracy of the transcriptomic data.

### Enrichment analysis

**Fig. 6:**
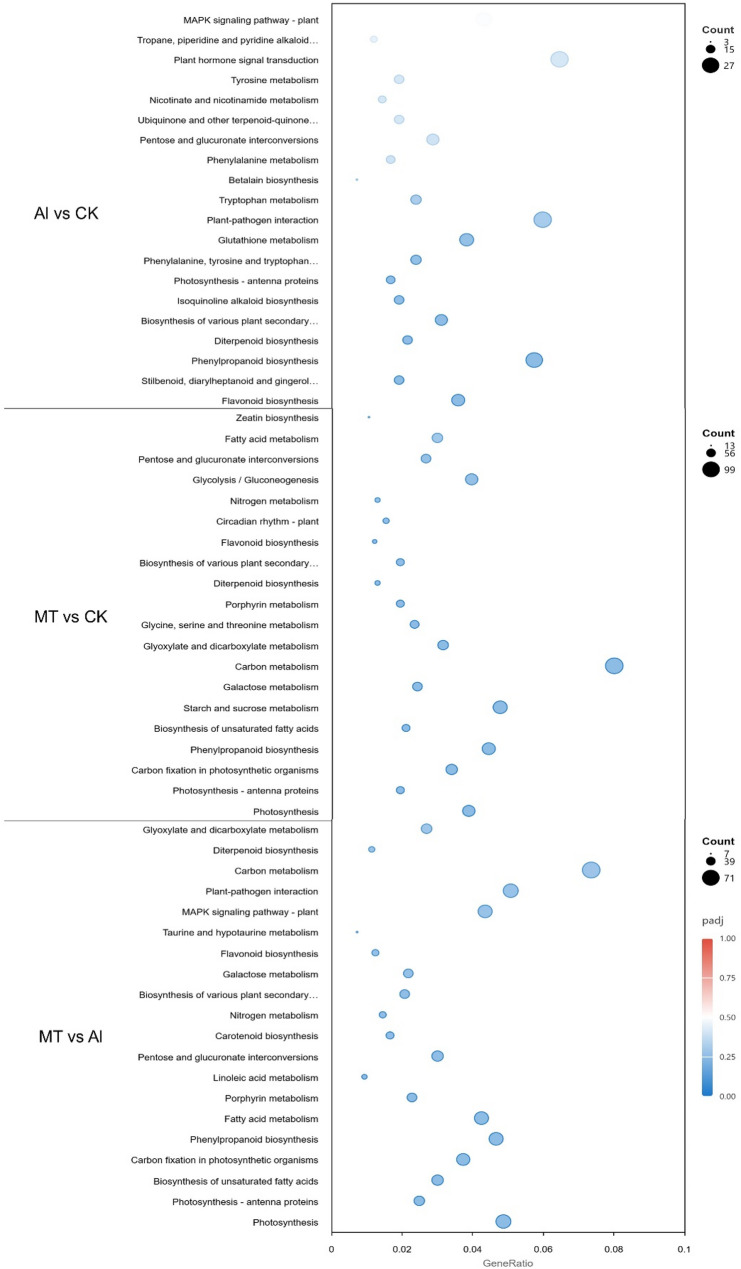
KEGG enrichment analysis of differentially expressed genes

KEGG enrichment analysis revealed distinct transcriptional responses to aluminum stress and MT treatment among the three comparison groups (Fig. 6). A total of 418, 1,237, and 965 differentially expressed genes were assigned to 116, 127, and 126 pathways in the Al vs. CK, MT vs. CK, and MT vs. Al comparisons, respectively. In the Al vs. CK comparison, differentially expressed genes were mainly enriched in secondary metabolite–related pathways, including flavonoid and phenylpropanoid biosynthesis, which are closely associated with antioxidant defense and stress adaptation. This enrichment suggests that aluminum stress induces metabolic adjustments aimed at alleviating oxidative damage. In contrast, the MT vs. CK comparison showed predominant enrichment of photosynthesis-related pathways, indicating that MT promotes photosynthetic activity and energy metabolism. Similarly, in the MT vs. Al comparison, photosynthesis-associated pathways remained significantly enriched, together with the biosynthesis of unsaturated fatty acids, suggesting that MT alleviates aluminum-induced impairment of photosynthetic processes and contributes to membrane stability under stress conditions.

**Fig. 7:**
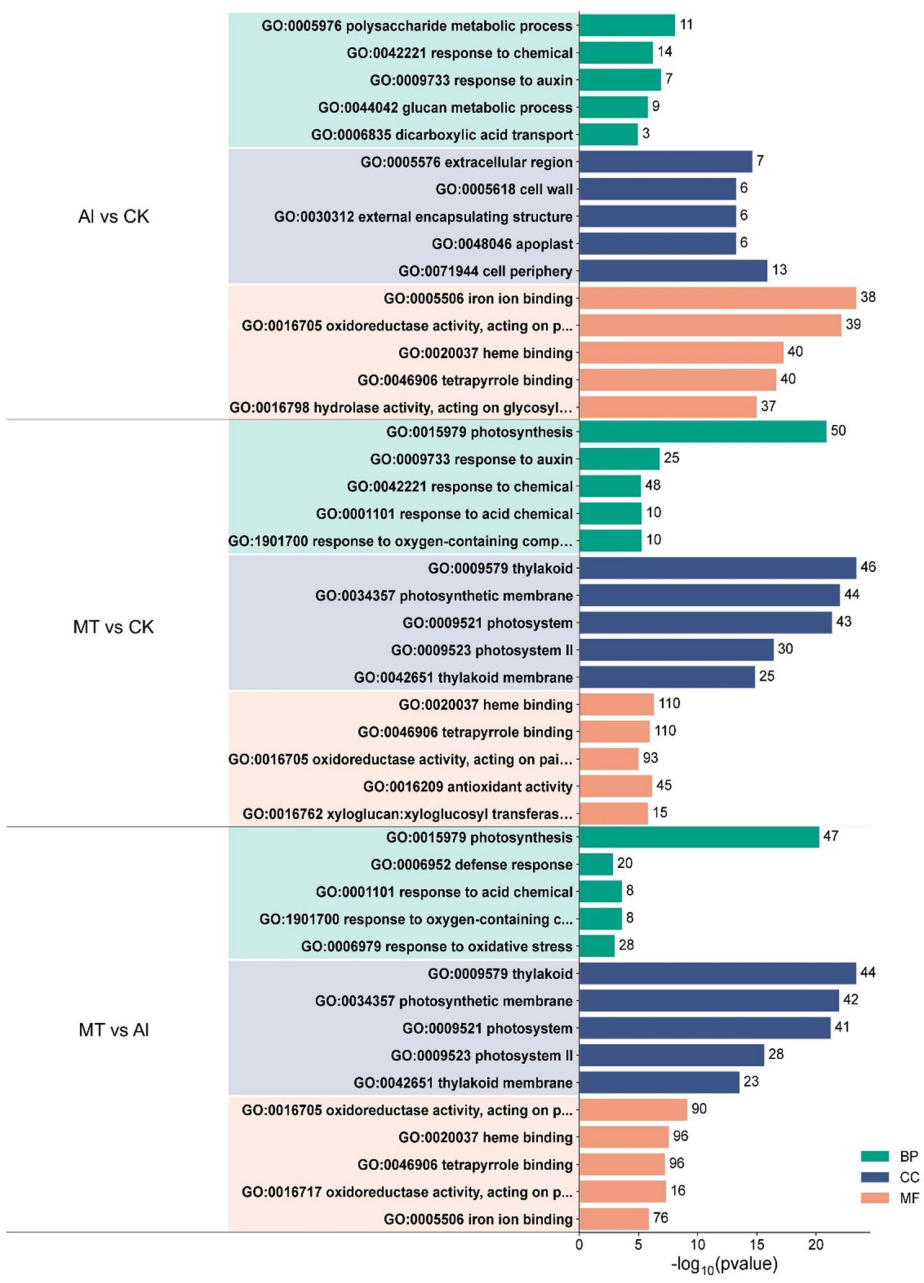
GO enrichment analysis of differentially expressed genes

GO enrichment analysis further illustrated functional differences associated with aluminum stress and MT treatment (Fig. 7). Significantly enriched GO terms varied among the three comparison groups, reflecting distinct regulatory responses. In the Al vs. CK comparison, enriched terms were mainly related to polysaccharide metabolism, the extracellular region, the cell wall, and iron ion binding, suggesting that aluminum stress affects cell wall–associated processes, metal homeostasis, and redox regulation. These changes may contribute to restricting aluminum toxicity and maintaining cellular stability. In the MT vs. CK comparison, photosynthesis-related terms, including photosynthesis, thylakoid, and photosystem, were predominantly enriched, together with oxidoreductase and antioxidant activities. This enrichment indicates that MT enhances photosynthetic capacity and redox balance under non-stress conditions. Similarly, in the MT vs. Al comparison, photosynthesis-associated terms and oxidoreductase activity remained highly enriched, suggesting that MT partially reverses aluminum-induced transcriptional inhibition of photosynthesis and reinforces antioxidant-related functions, thereby contributing to improved aluminum tolerance.

### Analysis of antioxidant metabolic pathways

**Fig. 8:**
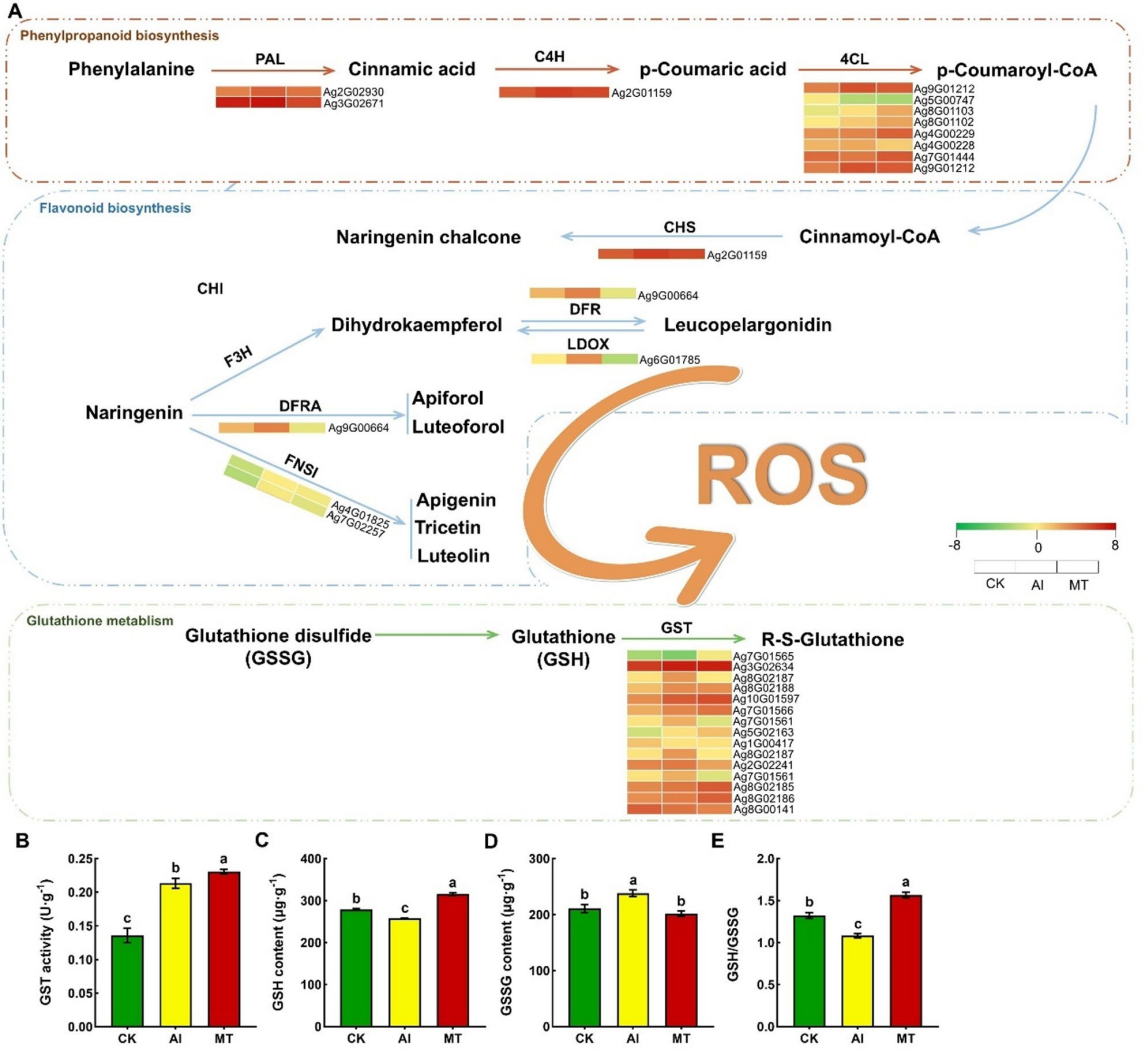
Changes in differentially expressed genes involved in antioxidant metabolic pathways. **A** Antioxidant metabolic pathways.**B** GST activity. **C** GSH content. **D** GSSG content. **E** GSH/GSSG

As shown in Fig. 8A, under the Al treatment, the expression of key enzymes in the phenylpropanoid biosynthesis pathway, including phenylalanine ammonia-lyase (*PAL*), cinnamic acid-4-hydroxylase (*C4H*), and several p-coumaroyl-CoA ligases (*4CLs*), was substantially upregulated. In the flavonoid biosynthesis pathway, the Al treatment markedly affected the expression of genes such as chalcone synthase (*CHS*) and dihydroflavonol 4-reductase (*DFR*), thereby regulating the synthesis of antioxidant flavonoid compounds, including dihydrokaempferol and apiforol. These changes contribute to enhanced antioxidant capacity, improved free radical scavenging, and improved metal chelation ability in plants. Under the MT treatment, the overall transcript abundance of genes associated with phenylpropanoid and flavonoid biosynthesis pathways were lower than those under the Al treatment, indicating that MT partially reduced the intensity of oxidative stress and cellular damage signaling. In the glutathione metabolism pathway, MT treatment resulted in the upregulation of most glutathione S-transferase (*GST*) genes, significantly promoting the synthesis of glutathione and R-S-glutathione conjugates, thereby further enhancing plant tolerance to aluminum stress. To verify changes in genes related to glutathione metabolism, glutathione-related parameters were measured, as shown in Fig. 8B–E. Compared with the CK, GSH content decreased, while GSSG content increased under Al treatment, resulting in a significant reduction in the GSH/GSSG ratio, whereas MT treatment exhibited the opposite trend. Meanwhile, GST activity reached the highest level under MT treatment, consistent with the transcriptome results. Overall, MT enhanced the antioxidant capacity of celery under aluminum stress by regulating these key metabolic pathways, reduced oxidative damage, and consequently alleviated the toxic effects of aluminum on plant growth.

### Analysis of hormone signal transduction pathways

**Fig. 9:**
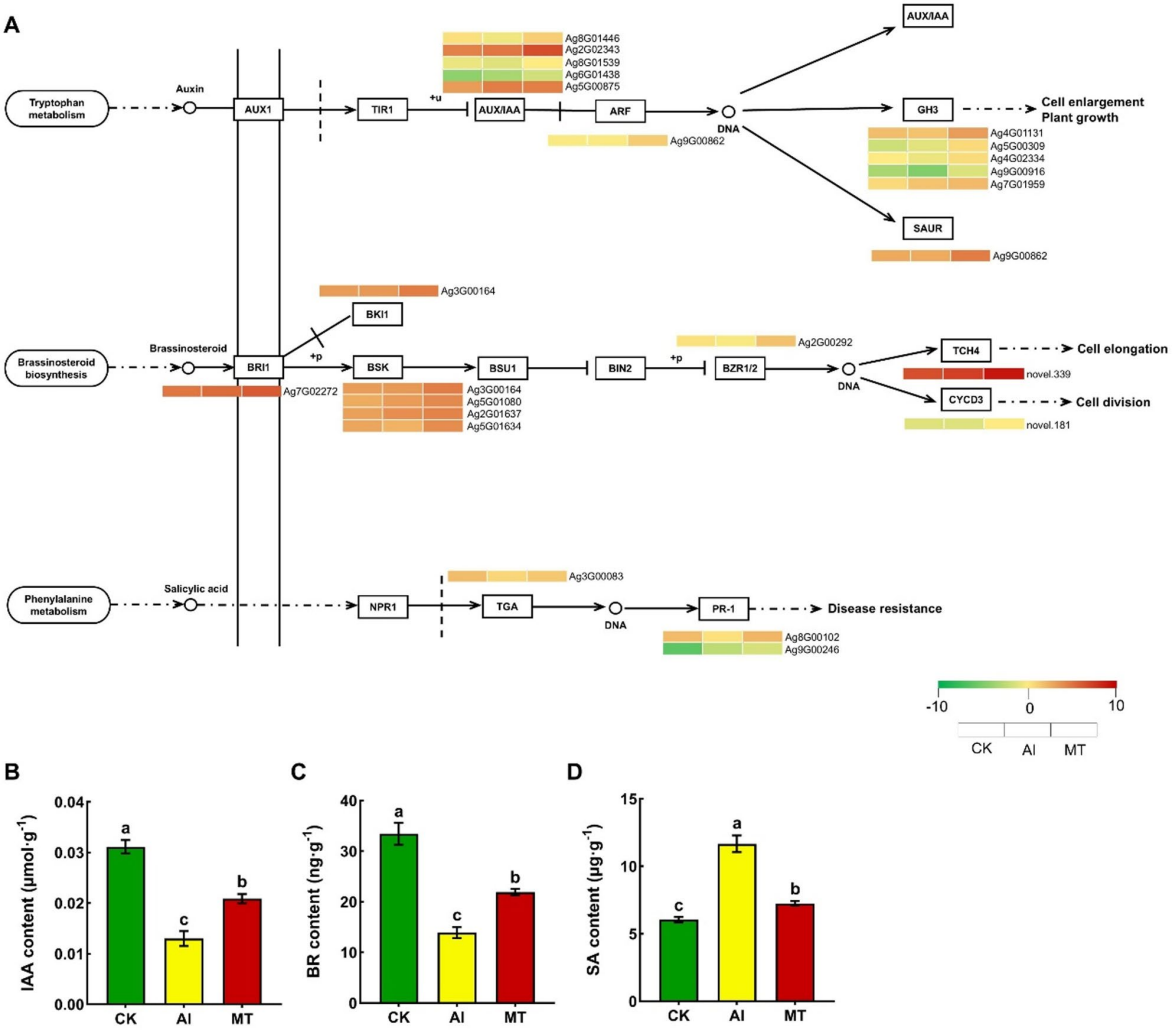
Differential expression patterns of genes associated with plant hormone signal transduction. **A** Hormone signal transduction pathways. **B** IAA content. **C** BR content. **D** SA content

Figure 9A depicts the expression profiles of genes involved in auxin, brassinosteroid, and salicylic acid signal transduction under different treatments. Overall, genes related to *AUX/IAA* and *ARF* in the auxin signaling pathway were generally upregulated under MT treatment, leading to further activation of auxin-responsive genes. Key early auxin-responsive genes, such as *GH3* and *SAUR*, were also notably enhanced under MT treatment, suggesting that MT facilitates the recovery and transmission of auxin signaling, promoting cell elongation and growth under aluminum stress. In the brassinosteroid signaling pathway, key node genes encoding the membrane receptor *BRI1* and its regulatory factors *BKI1* and *BSK* exhibited overall higher expression under MT treatment. In addition, the downstream transcription factors *BZR1/2* and their target genes *TCH4* and *CYCD3* exhibited substantially higher expression levels under MT treatment compared with the Al treatment. This suggests that MT enhanced the efficiency of BR signal transduction to growth-related genes, thereby promoting cell elongation and cell division. For the salicylic acid (SA) signaling pathway, MT treatment increased the expression of *TGA* and the defense marker gene *PR-1* compared to Al treatment, suggesting that MT activates SA-dependent defense signaling. The contents of related hormones were further measured as auxiliary indicators, as shown in Figs. 9B–D, under aluminum stress, the contents of IAA and BR showed a declining trend, whereas they exhibited a certain degree of recovery under MT treatment. However, the content of SA exhibited an opposite trend to that of IAA and BR across the different treatments.Overall, MT synchronously enhanced key genes across multiple hormone pathways, including auxin, brassinosteroid, and salicylic acid signaling, providing a molecular basis for subsequent cell growth, division, and stress resistance responses.

## Discussion

### MT alleviates aluminum-induced growth inhibition in celery

For many plant species, aluminum stress primarily affects root growth [33], prolonged exposure to aluminum stress leads to a reduction in lateral root formation and root cap detachment, which severely impairs the absorption of water and nutrients and ultimately results in a reduced crop yields. Therefore, reducing the root uptake of Al^3+^ is considered a key factor in alleviating aluminum toxicity [34]. The results of this study revealed that aluminum stress suppressed celery growth, especially in terms of root morphology and biomass accumulation, these are consistent with other research findings [35]. After the application of exogenous MT, the root morphology and biomass accumulation of celery were significantly improved. This suggests that MT can effectively alleviate the inhibitory effects of aluminum stress on root growth and promote overall plant growth, which is consistent with the findings of Zhang [36]. In addition, MT markedly enhanced root activity, showing a 250.9% increase compared to the Al treatment. This indicates that MT effectively alleviated the damage caused by aluminum stress to root metabolism [37]. Notably, although CK exhibited the most favorable root morphology, its fresh weight was lower than that observed under MT treatment. This may be attributed to MT enhancing celery root activity, thereby promoting water uptake and retention and ultimately increasing overall biomass, consistent with the findings of Ubaidillah [38]. Changes in Al^3+^ content further supported these results. Aluminum stress significantly enhanced aluminum accumulation in different parts of celery, including leaves, petioles, and roots. By contrast, treatment with exogenous MT led to a significant reduction in aluminum concentrations across all tissues, with decreases of 39.5% in leaves, 37.5% in petioles, and 37.8% in roots. These findings demonstrate that MT can limit aluminum accumulation and thereby alleviate aluminum-induced damage. The potential mechanism may be related to melatonin-mediated regulation of Al^3+^ uptake and distribution. On the one hand, melatonin may enhance the external immobilization and chelation of Al^3+^ by influencing cell wall components or promoting organic acid secretion, thereby reducing its entry into cells [39]. On the other hand, melatonin may regulate the expression of related transporter proteins, thereby facilitating the compartmentalization or sequestration of aluminum within cells and decreasing its accumulation in plants [40]. However, these mechanisms still require further experimental validation.

These results indicate that MT treatment enhances root morphology, root activity, and biomass accumulation in celery under aluminum stress, accompanied by altered Al^3+^ uptake, suggesting that melatonin may partially mitigate the adverse effects of aluminum stress on celery growth.

### MT alleviates aluminum-induced inhibition of photosynthesis in celery

Light is an indispensable environmental factor that regulates plant physiological and metabolic processes primarily through photosynthesis [41]. Aluminum toxicity disrupts chloroplast structure, leading to deformation and distortion of thylakoid membranes, reduced content and stability of photosynthetic pigments, and ultimately inhibition of photosynthetic efficiency [42]. In this study, aluminum treatment significantly suppressed photosynthesis in celery, as evidenced by decreases in Tr, Gs, and Pn. After the application of exogenous MT, Tr and Gs increased significantly by 144.5% and 70.5%, respectively, compared with the Al treatment, and Pn recovered to a level comparable to that of the control. Furthermore, aluminum stress markedly reduced chlorophyll fluorescence parameters of celery, indicating impairment of PSⅡ function. In contrast, MT treatment significantly improved these parameters, suggesting that MT helps maintain PSⅡ photochemical activity and electron transport capacity [43]. Aluminum stress also significantly decreased the contents of photosynthetic pigments in celery leaves, indicating that Al^3+^ disrupted pigment accumulation. After MT treatment, pigment contents were significantly increased; although they did not fully recover to control levels, they were substantially higher than those under aluminum stress alone, demonstrating a regulatory effect of MT on photosynthetic pigment biosynthesis. This phenomenon aligns with findings from other studies indicating that MT can elevate photosynthetic pigment levels and photosynthetic efficiency in plants under stress conditions [44–45].

In summary, this present study reveals that exogenous MT effectively counteracts aluminum-induced inhibition of photosynthesis in celery by improving photosynthetic physiological traits, enhancing PSⅡ performance, and promoting the accumulation of photosynthetic pigments. These results provide important physiological evidence supporting the application of MT in mitigating metal-induced stress in plants.

### MT enhances antioxidant defense and redox homeostasis under aluminum stress

ROS are highly reactive oxygen-containing molecules continuously produced during aerobic metabolism in plant cells, playing a crucial role in signal transduction and stress responses [46]. While ROS act as key signaling mediators that integrate environmental cues and activate defense pathways, an imbalance between their production and removal under stress may induce oxidative damage at the cellular level [47]. The results of this study show that Al treatment markedly increased the levels of ROS and MDA, indicating that Al^3+^ triggered oxidative stress responses and disrupted the antioxidant balance in plants [48], thereby exacerbating oxidative damage. Aluminum stress also enhanced the activities of antioxidant enzymes, suggesting that plants attempt to cope with excessive ROS by upregulating antioxidant defenses [49]. However, despite the increased antioxidant enzyme activities under aluminum stress, oxidative damage remained severe, likely because the rate of ROS generation exceeded the scavenging capacity [50]. After the application of exogenous MT, oxidative damage in celery was markedly alleviated, while antioxidant enzyme activities were further enhanced under MT treatment. These findings suggest that MT strengthens the plant antioxidant defense capacity and thereby effectively reduces Al^3+^-induced oxidative damage [51]. This effect not only improves antioxidant capacity but also indirectly protects the photosynthetic apparatus, promoting the recovery of plant growth [52]. The antioxidant properties of MT confer enhanced stress tolerance in plants, highlighting its important role in alleviating aluminum stress.

The results indicate that melatonin can effectively alleviate aluminum stress–induced oxidative damage by enhancing the plant antioxidant defense system and maintaining intracellular redox homeostasis, thereby promoting plant growth recovery and playing an important role in improving plant tolerance to aluminum stress.

### MT modulates antioxidant metabolic pathways to alleviate aluminum stress

In plants, adverse conditions such as aluminum stress usually induce excessive accumulation of ROS, if ROS are not effectively scavenged, they can cause protein oxidation and cellular structural damage [53]. To counteract such challenges, plants have developed complex, multilayered antioxidant defense mechanisms, among which the phenylpropanoid biosynthesis pathway, flavonoid biosynthesis pathway, and glutathione metabolism pathway are key components in responses to abiotic stress. The phenylpropanoid pathway begins with *PAL* as the key enzyme, regulating the synthesis of various phenolic compounds that play important roles in plant stress responses. Previous systematic reviews have shown that phenylpropanoid metabolites possess dual functions in antioxidant defense and structural protection under stress conditions, making this pathway a crucial adaptive metabolic route [54]. In addition, flavonoids, flavonols, and their derivatives produced through the flavonoid biosynthesis pathway exhibit strong free radical–scavenging and metal-chelating capacities, providing significant antioxidant protection under heavy metal stress [55–56]. GSH, a major non-enzymatic antioxidant in plant cells, plays a key role in the interaction between ascorbate and glutathione in the cycle, directly scavenging hydrogen peroxide and maintaining cellular redox homeostasis [57]. Moreover, numerous studies have demonstrated that upregulation of *GST* genes is closely associated with enhanced stress tolerance, particularly under metal stress, where GST-mediated detoxification reactions can markedly reduce oxidative damage [58–59].

Under aluminum stress, genes involved in phenylpropanoid and flavonoid biosynthesis (such as *PAL*, *C4H*, *4CL*, and *CHS*) were generally upregulated, indicating that celery activated a defense program centered on phenolic and flavonoid secondary metabolism. These metabolites can not only directly scavenge reactive oxygen species but also mitigate stress damage through mechanisms such as metal ion chelation, protection of membrane systems, and reinforcement of cell walls. Therefore, they are often regarded as an important “non-enzymatic antioxidant barrier” for plants in response to stress-induced oxidative damage [60], and this finding is consistent with previous reports showing that plants often upregulate phenylpropanoid and flavonoid pathways under metal stress to compensate for oxidative damage caused by excessive ROS accumulation [61]. Under MT treatment, the expression levels of genes associated with these pathways were reduced, which may suggest that melatonin application alleviated oxidative stress intensity, thereby decreasing the plant’s reliance on energy-intensive secondary metabolic pathways, this observation supports the notion that melatonin exerts negative feedback regulation on secondary metabolism by reducing ROS levels [62]. Meanwhile, the possibility that melatonin may reprogram plant antioxidant strategies by modulating the balance among different antioxidant systems cannot be excluded. Conversely, genes involved in glutathione metabolism and the *GST* family showed higher expression levels under MT treatment. Mechanistically, the upregulation of GST is of considerable significance. GST can not only catalyze the conjugation of glutathione with reactive electrophilic molecules or lipid peroxidation products to reduce oxidative damage but also participate in the binding and transport of anthocyanins and other flavonoid compounds. Therefore, the upregulation of GST-related genes and the enhancement of their physiological activity may indicate that melatonin not only improves GSH-dependent detoxification efficiency but also promotes the transport of stress-related secondary metabolites [63]. The higher GSH content and GSH/GSSG ratio observed under MT treatment are consistent with previous findings that melatonin enhances plant stress resistance by increasing GSH levels and maintaining a high GSH/GSSG ratio [64]. Maintaining a larger GSH pool and a more favorable GSH/GSSG status contributes to protein stabilization [65], protection of chloroplast function [66], and effective suppression of continuous ROS generation.

In summary, under aluminum stress, plants primarily rely on phenylpropanoid and flavonoid pathways to enhance antioxidant defense, whereas MT application refines the antioxidant metabolic strategy by not only effectively reducing ROS levels but also decreasing the energy cost associated with secondary metabolism. This provides a molecular basis for maintaining growth and physiological homeostasis under aluminum stress and further elucidates the potential mechanisms underlying MT-mediated alleviation of aluminum stress.

### MT modulates hormone signal transduction pathways to alleviate aluminum stress

Plant hormone signaling pathways serve as central regulators of stress response processes by sensing environmental changes and coordinating growth and defense processes, thereby enabling plants to maintain physiological balance under adverse conditions [67]. Recent studies have shown that aluminum stress not only triggers oxidative stress directly but also interferes with multiple hormone signaling pathways, leading to the inhibition of cell elongation, cell division, and root growth. As a small molecule with both antioxidant and signaling regulatory functions, MT has been demonstrated to interact with various plant hormone pathways, thereby balancing plant growth and stress resistance under under stress conditions [68–69].

Under aluminum stress, enhanced auxin (AUX) signaling is often observed in the root apex transition and meristematic zones, making the auxin pathway a key hub linking stress signals to growth regulation [70]. The *GH3* family modulates endogenous auxin levels by conjugating free IAA with amino acids, thereby contributing to auxin homeostasis under stress conditions [71–72]. Meanwhile, *SAUR* genes are closely associated with cell elongation, mainly by regulating H^+^-ATPase activity to drive cell expansion [73]. In this study, melatonin treatment affected the expression of key genes in the auxin signaling pathway, including *AUX/IAA*, *GH3*, and *SAUR*, suggesting that it may alleviate the disruption of auxin signaling caused by aluminum stress, thereby contributing to the restoration of cell elongation–related growth processes. Previous studies have also demonstrated that heavy metal stress can interfere with auxin signaling, whereas melatonin can mitigate this inhibitory effect by modulating the hormonal network [74]. Meanwhile, genes associated with the brassinosteroid signaling pathway (such as *TCH4* and *CYCD3*) exhibited an upregulated trend under MT treatment, indicating that melatonin may participate in growth regulation by influencing BR signaling, thereby alleviating growth inhibition induced by aluminum stress [75]. *TCH4* (*XTH22*), a member of the xyloglucan endotransglucosylase/hydrolase (*XTH*) family [76], by regulating arabinoxylan content, the cell wall’s binding capacity for Al^3+^ can be reduced. In this study, the significant decrease in Al^3+^ content across various tissues under MT treatment may be associated with this mechanism [77]. The increased expression of *TCH4* observed under MT treatment shows that enhanced BR signaling promotes growth-related signals, enabling celery tissues to more readily restore expansive growth through cell wall restructuring. In contrast to cell wall–level regulation, *CYCD3* functions as a key regulator of cell division in the BR pathway, previous studies have demonstrated that BR can induce *CYCD3* transcription and promote cell division, playing a crucial role in G1/S transition and proliferation maintenance [78]. The *CYCD3* subfamily is recognized as a central node linking cell proliferation to organ growth and is closely associated with tissue differentiation [79]. Therefore, the upregulation of *CYCD3* under MT treatment suggests that MT may help maintain high cell cycle activity in proliferative tissues such as root apices and vascular tissues by enhancing BR signaling, thereby alleviating aluminum-induced growth arrest. Notably, exogenous BR has been shown to mitigate growth damage caused by aluminum toxicity in rice [80]. Combined with the significant upregulation of *TCH4* (cell wall remodeling) and *CYCD3* (cell division) under MT treatment, these findings support the hypothesis that MT promotes growth recovery in celery under aluminum stress by activating BR-mediated growth signaling, enhancing cell wall plasticity, and facilitating cell cycle progression. The salicylic acid (SA) signaling pathway, an important regulator of antioxidant processes, mainly relies on the *NPR1–TGA* transcriptional regulatory module. Among them, *TGA*-type bZIP transcription factors function as critical intermediaries in salicylic acid–mediated defense signaling [81–82] can activate a range of defense-associated genes, including *PR-1*. Meanwhile, the expression level of *PR-1* is often used to reflect the activation intensity of SA signaling and is closely associated with plant stress resistance [83]. It is noteworthy that the content of SA exhibited an opposite trend compared to that of IAA and BR under different treatments. This may be due to the antagonistic relationship between auxin signaling and salicylic acid signaling, consistent with the findings of Kong [84]. Therefore, the *TGA* and *PR-1* upregulation observed under MT treatment in this study indicates that MT may enhance antioxidant defense capacity by modulating SA signaling intensity and activating the downstream *TGA–PR-1* module, thereby helping celery maintain physiological balance under aluminum stress.

**Fig. 10:**
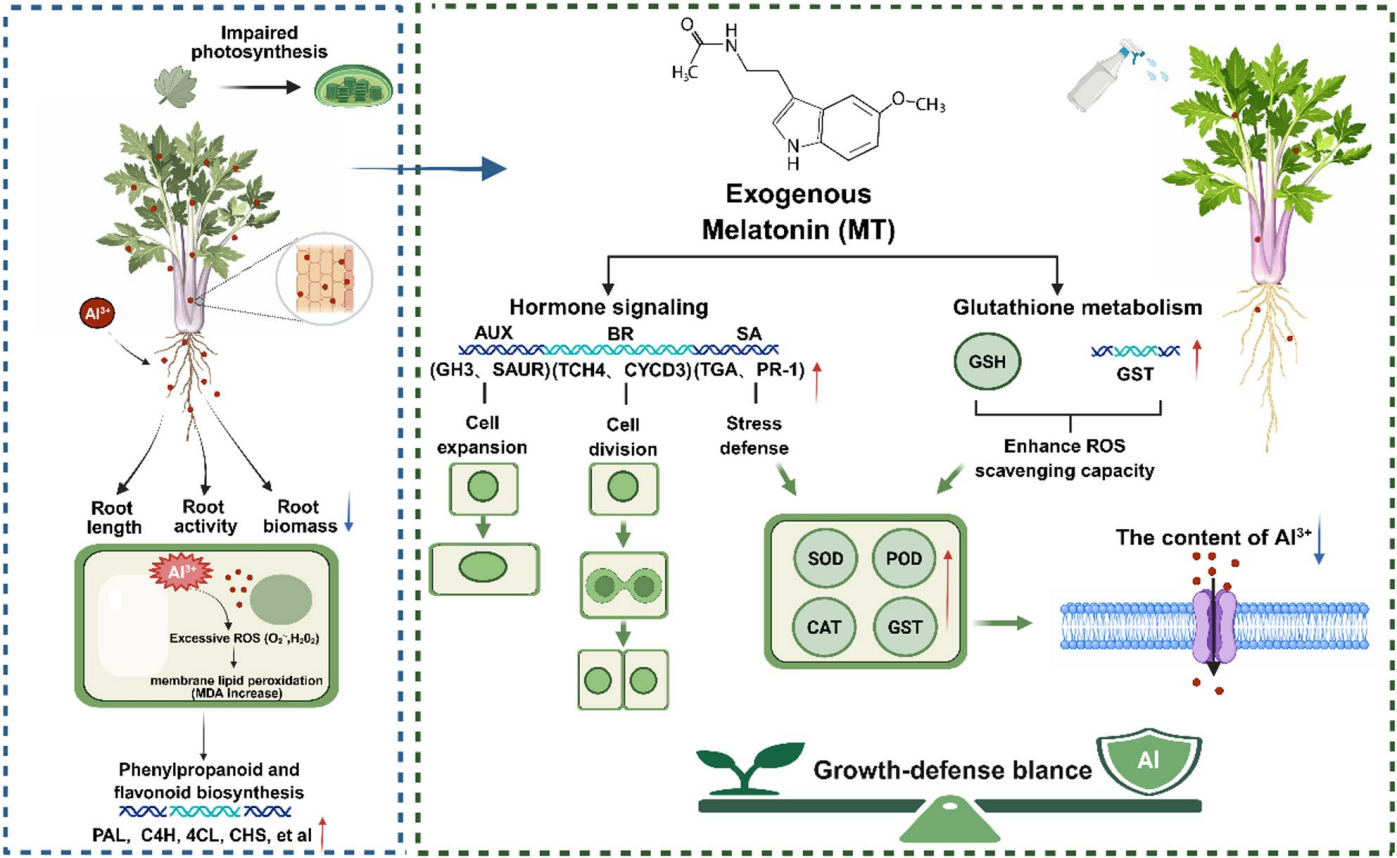
Schematic diagram of the effects of exogenous melatonin on internal physiological metabolism in celery under aluminum stress

Overall, melatonin treatment affected the expression of genes associated with multiple hormone signaling pathways, suggesting its involvement in regulating growth- and defense-related processes under aluminum stress. This regulatory effect may be associated with the promotion of cell elongation and division, as well as alterations in the antioxidant system, thereby contributing to the maintenance of intracellular redox homeostasis and facilitating the alleviation of aluminum toxicity and the recovery of plant growth (Fig. 10).

## Conclusions

This study suggests that melatonin treatment may enhance the adaptability of celery to aluminum stress by influencing processes related to antioxidant metabolism and plant hormone signaling pathways, thereby providing a theoretical basis for the regulation of aluminum tolerance in crops. However, this research primarily focuses on transcriptomic-level analysis, and the key genes involved in MT-regulated aluminum tolerance and their mechanisms still need to be further clarified through functional validation.

## Supplementary Information

Supplementary Material 1.

## Data Availability

The data that support the results are included within the article and supplement material.
