# Supplementary material for: Integrated physiological and transcriptomic analyses reveal MT-mediated aluminum tolerance mechanisms in celery
Source: BMC Plant Biol. 2026 Jun 3;26:1331. doi: 10.1186/s12870-026-08994-w (PMC13449324; doi:10.1186/s12870-026-08994-w)
Supplement: Supplementary file 1 [file 12870_2026_8994_MOESM1_ESM.zip › Supplementary Information/Supplementary Material 1..docx]

Figure 1 and Table 1 present the results of the aluminum concentration screening experiment. Figure 1 illustrates the effects of different aluminum concentrations on celery. Based on various physiological and biochemical parameters combined with membership function analysis, the results indicate that 1600 μmol·L^-1^ exhibited the strongest toxic effect, followed by 800 μmol·L^-1^. However, celery plants subjected to 1600 μmol·L⁻¹ experienced excessive toxicity, making them unsuitable for subsequent alleviation experiments. Therefore, 800 μmol·L^-1^ was selected as the aluminum concentration for the experiments.

**Figure 1. Effect of different aluminum treatments on the growth status of celery.** After 20 d of acclimation under hydroponic conditions, predetermined amounts of aluminum sulfate octadecahydrate [Al_2_(SO_4_)_3_·18H_2_O] were added to the nutrient solution. The solution was renewed every 5 d, with 8 L replaced each time, for a total of three times. The experiment was conducted using a completely randomized design with 12 replicates per treatment. A total of five treatments were established: 0, 200, 400, 800, and 1600 μmol·L^-1^.

**Table 1. Principal component scores, composite scores, and rankings under different aluminum treatments.**

| Indicator classification | Treatment | U_1_ | U_2_ | D value | Rank |
| --- | --- | --- | --- | --- | --- |
|  |  |  |  |  |  |
| Leaf growth  physiology | CK | 0.674 | 0.000 | 0.573 | 3 |
|  | 200 | 0.825 | 0.328 | 0.750 | 2 |
|  | 400 | 1.000 | 1.000 | 1.000 | 1 |
|  | 800 | 0.000 | 0.722 | 0.108 | 4 |
|  | 1600 | 0.006 | 0.518 | 0.083 | 5 |
|  | Weight (W_i_) | 0.850 | 0.150 |  |  |
| Petiole  growth  physiology | CK | 0.693 | 0.000 | 0.603 | 3 |
|  | 200 | 0.870 | 0.139 | 0.775 | 2 |
|  | 400 | 1.000 | 1.000 | 1.000 | 1 |
|  | 800 | 0.054 | 0.537 | 0.117 | 4 |
|  | 1600 | 0.000 | 0.543 | 0.071 | 5 |
|  | Weight (W_i_) | 0.870 | 0.130 |  |  |

Figure 2 and Table 2 present the results of the melatonin concentration screening experiment. Figure 2 illustrates the alleviative effects of different melatonin concentrations on celery under aluminum stress. Based on various physiological and biochemical parameters combined with membership function analysis, the results indicate that 150 μmol·L^-1^ MT exhibited the optimal effect. Therefore, 150 μmol·L^-1^ was selected as the melatonin concentration for the experiments.

**Figure 2. Effect of different MT on the growth status of celery under aluminum stress.** After 20 d of acclimation, leaves were sprayed with melatonin solutions of different concentrations until runoff. The application was performed every 5 d, and Al treatment was applied simultaneously at the same interval. The experiment was conducted using a completely randomized design with six treatments, each comprising 12 replicates: CK (0 μmol·L^-1^ MT + 0 μmol·L^-1^ Al^3+^), Al (0 μmol·L^-1^ MT + 800 μmol·L^-1^ Al^3+^), (100 μmol·L^-1^ MT + 800 μmol·L^-1^ Al^3+^), (150 μmol·L^-1^ MT + 800 μmol·L^-1^ Al^3+^), (200 μmol·L^-1^ MT + 800 μmol·L^-1^ Al^3+^), and (250 μmol·L^-1^ MT + 800 μmol·L^-1^ Al^3+^).

**Table 2. Principal component scores, comprehensive scores, and rankings under different MT treatments**

| Indicator classification | | Treatment | U_1_ | U_2_ | U_3_ | D value | Rank |  |
| --- | --- | --- | --- | --- | --- | --- | --- | --- |
|  |  |  |  |  |  |  |  |  |
| Leaf growth physiology | CK | | 1.000 | 0.710 | 0.000 | 0.835 | 2 |  |
|  | Al | | 0.000 | 1.000 | 0.401 | 0.197 | 6 |  |
|  | 100 | | 0.340 | 0.000 | 0.370 | 0.294 | 5 |  |
|  | 150 | | 0.945 | 0.708 | 1.000 | 0.917 | 1 |  |
|  | 200 | | 0.563 | 0.494 | 0.683 | 0.568 | 3 |  |
|  | 250 | | 0.414 | 0.538 | 0.946 | 0.497 | 4 |  |
|  | Weight (W_i_) | | 0.730 | 0.148 | 0.122 |  |  |  |
| Petiole growth physiology | CK | | 1.000 | 0.748 | 1.000 | 0.963 | 1 |  |
|  | Al | | 0.000 | 1.000 | 0.589 | 0.213 | 6 |  |
|  | 100 | | 0.303 | 0.000 | 0.714 | 0.303 | 5 |  |
|  | 150 | | 0.988 | 0.610 | 0.002 | 0.825 | 2 |  |
|  | 200 | | 0.523 | 0.399 | 0.377 | 0.489 | 3 |  |
|  | 250 | | 0.351 | 0.578 | 0.000 | 0.347 | 4 |  |
|  | Weight (W_i_) | | 0.742 | 0.149 | 0.109 |  |  |  |

**Table 3. Effect of different MT on the biomass of celery under aluminum stress**

| Treatment | Fresh weigh/g | | Dry weigh/g | |
| --- | --- | --- | --- | --- |
|  | Shoot | Root | Shoot | Root |
| CK | 35.17±0.08 b | 11.60±0.45 b | 3.88±0.11 b | 1.02±0.04 b |
| Al | 28.49±0.44 d | 8.95±0.28 c | 3.15±0.09 e | 0.80±0.02 d |
| 100 | 31.59±0.54 c | 8.09±0.18 d | 3.49±0.11 cd | 0.69±0.02 e |
| 150 | 37.84±0.92 a | 12.95±0.39 a | 4.59±0.10 a | 0.87±0.03 c |
| 200 | 31.73±0.84 c | 9.66±0.39 c | 3.59±0.06 c | 1.08±0.03 a |
| 250 | 32.01±0.69 c | 9.71±0.20 c | 3.38±0.05 d | 0.78±0.02 d |

**Figure 3. Effect of different MT on the root vitality of celery under aluminum stress**
