# Supplementary material for: Integrated physiological and transcriptomic analyses reveal MT-mediated aluminum tolerance mechanisms in celery
Source: BMC Plant Biol. 2026 Jun 3;26:1331. doi: 10.1186/s12870-026-08994-w (PMC13449324; doi:10.1186/s12870-026-08994-w)
Supplement: Supplementary file 1 [file 12870_2026_8994_MOESM1_ESM.zip › Supplementary Information/Supplementary Material 4..docx]

Supplementary Material 4. (A) Principal Component Analysis Results Plot. (B) Sample Correlation Heatmap.
