# Supplementary material for: Integrated physiological and transcriptomic analyses reveal MT-mediated aluminum tolerance mechanisms in celery
Source: BMC Plant Biol. 2026 Jun 3;26:1331. doi: 10.1186/s12870-026-08994-w (PMC13449324; doi:10.1186/s12870-026-08994-w)
Supplement: Supplementary file 1 [file 12870_2026_8994_MOESM1_ESM.zip › Supplementary Information/Supplementary Material 5.zip!/Supplementary Material 5/Supplementary Figure 5..docx]

**Supplementary Material 5. KEGG enrichment analysis of unique genes in each comparison group. (A) KEGG enrichment analysis diagram for CK vs. Al. (B) KEGG enrichment analysis diagram for MT vs. CK. (C) KEGG enrichment analysis diagram for MT vs. Al.**
